# Effective Antibacterial/Photocatalytic Activity of ZnO Nanomaterials Synthesized under Low Temperature and Alkaline Conditions

**DOI:** 10.3390/nano12244417

**Published:** 2022-12-11

**Authors:** Sujeong Kim, Hyerim Park, Sadanand Pandey, Daewon Jeong, Chul-Tae Lee, Jeong Yeon Do, Sun-Min Park, Misook Kang

**Affiliations:** 1 Department of Chemistry, College of Natural Sciences, Yeungnam University, Gyeongsan 38541, Republic of Korea; 2 Department of Microbiology, Laboratory of Bone Metabolism and Control, Yeungnam University College of Medicine, Daegu 42415, Republic of Korea; 3 Industry-University Cooperation Foundation, Dongduk Women’s University, Seoul 02748, Republic of Korea; 4 Korea Evaluation Institute of Industrial Technology (Keit), Cheomdan-Ro 8-Gil, Dong-Gu, Daegu 41069, Republic of Korea; 5 Korea Institute of Ceramic Engineering and Technology (KICET), Jinju 52851, Republic of Korea

**Keywords:** ZnO nanomaterials, low temperature, alkaline condition, antibacterial, photocatalytic activity

## Abstract

The purpose of this study was to evaluate the surface properties of ZnO nanomaterials based on their ability to photodegrade methyl blue dye (MB) and to show their antibacterial properties against different types of Gram-positive bacteria (*Bacillus manliponensis*, *Micrococcus luteus*, *Staphylococcus aureus)* and Gram-negative bacteria (*Escherichia coli)*. In this study, ZnO nanomaterials were synthesized rapidly and easily in the presence of 1–4 M NaOH at a low temperature of 40 °C within 4 h. It was found that the ZnO nanomaterials obtained from the 1.0 M (ZnO–1M) and 2.0 M (ZnO–2M) aqueous solutions of NaOH had spherical and needle-shaped forms, respectively. As the concentration of NaOH increased, needle thickness increased and the particles became rod-like. Although the ZnO nanomaterial shapes were different, the bandgap size remained almost unchanged. However, as the NaOH concentration increased, the energy position of the conduction band shifted upward. Photo current curves and photoluminescence intensities suggested that the recombination between photoexcited electrons and holes was low in the ZnO–4M materials prepared in 4.0 M NaOH solution; however, charge transfer was easy. ∙O_2_^−^ radicals were generated more than ∙OH radicals in ZnO–4M particles, showing stronger antibacterial activity against both Gram-positive and Gram-negative bacteria and stronger decomposition ability on MB dye. The results of this study suggest that on the ZnO nanomaterial surface, ∙O_2_^−^ radicals generated are more critical for antibacterial activity than particle shape.

## 1. Introduction

A good antibacterial material should have an ideal level of biocompatibility, antibacterial activity, and stability in a variety of environments and also be less susceptible to developing resistance to microorganisms, safe both in vivo and in vitro, and remain harmless to the environment after use [1]. Nanomaterials based on metals and their oxides have recently received substantial attention as antibacterial agents. Nanomaterials such as Ag, Au, and Cu possess antibacterial properties and exhibit surface plasmon resonance (SPR) [2,3,4]. In recent years, several metal oxides and carbon nanomaterials exhibiting semiconducting properties have been added to this group of antibacterial materials, including Fe_2_O_3_, ZnO, TiO_2_, CuO, CdO, Sn_2_O, Ce_2_O, MgO, Zr_2_O and carbon quantum dots (CQD) [5,6,7,8,9,10,11,12,13,14]. The interaction of these nanomaterials with bacterial species leads to the generation and accumulation of reactive oxygen species (ROS) such as singlet oxygen, hydroxyl radicals, hydrogen peroxide, and superoxide anions [15,16]. Therefore, the mechanism behind the antibacterial activities of the aforementioned nanomaterials depends on the ability of ROS to damage the internal components of bacteria, such as proteins and DNA [17], ultimately killing the microorganism.

Among these, an interesting substance is zinc oxide (ZnO) a II-VI semiconductor with a wide band gap energy (3.3 eV) and high excitation energy (60 eV) [18], which acts as an essential element with an important effect on biological entities. ZnO has attracted much attention because of its unique properties, such as various morphologies, large surface area to volume ratio, strong antibacterial activity, and biocompatibility [19]. Aside from its antibacterial and anti-inflammatory properties, it is also known to have diabetic, antifungal and antitumor properties that are helpful for wounds, hemorrhoids, eczema, and abrasions [20]. Hence, it is widely used in cosmetic products. Additionally, ZnO is an indispensable substitute material for the marine industry due to its activity against biofilm formation, since tin has recently been banned, and is recommended for buoys and ships as an antifouling material [21]. Zinc is a strong reducing element and is easily oxidized to form zinc oxide. However, ZnO is considered almost non-toxic to the human body [22]. Of course, there are reports that Zn^2+^ ions, which are free zinc ions, can harm cells [23].

Furthermore, nanostructured ZnO can have various shapes and properties, and the antibacterial and optical activities are different depending on the shape and size. In general, antimicrobial evaluations have shown that Gram-negative bacteria are less sensitive to metal oxide nanomaterials than Gram-positive bacteria. This is because of the characteristics of the cell wall structure of Gram-negative bacteria. Unlike that in Gram-positive bacteria, the cell wall of Gram-negative bacteria has an additional outer membrane that contains lipopolysaccharide (LPS). LPS enhances the barrier properties of the outer membrane and increases bacterial resistance. However, ZnO nanomaterials are known to act effectively against both Gram-negative and Gram-positive bacteria to inhibit their growth. Thus, ZnO nanomaterials possess antibacterial properties that are highly interesting and have unlimited potential for development in the biotechnology field. Moreover, a method for the simple mass production of stable ZnO nanomaterials with excellent performance at low temperatures is very useful from an industrial perspective. As shown in Table 1 below, ZnO crystallizes through a chemical reaction at a relatively high temperature and for a long period of time [24,25,26,27,28].

In the current article, we have developed a simple and facile ZnO synthesis at low temperature utilizing strong alkaline conditions. By adding sodium hydroxide (NaOH) as a capping agent (strong alkali), it is possible to synthesize shape controlled ZnO NPs with short reaction time (4 h) at a low temperature of 40 °C. The synthesized ZnO NPs have considerable properties. This structure is thermodynamically stable in the ambient environment. As a result, we were able to synthesize ZnO with a thermodynamically stable structure in the ambient environment without an additional sintering process with an initial low synthesis temperature. From an industrial standpoint, this simple method produces stable ZnO nanomaterials that exhibit excellent performance at low temperatures. Moreover, we employed hydrothermal synthesis that uses deionized water as a solvent at low temperatures, but in several previously reported articles, oxalic acid [26,27], which is considered toxic and hazardous, is used as a solvent and additive at high temperatures to improve crystallinity and shape control, for example, dichloromethane [27], ethylenediamine [28], etc. 

This study aimed to develop a low-temperature hydrothermal synthesis method in which ZnO nanoparticles are partially dissolved in a strong base in a molar ratio and spread out like a sheet only through the use of an alkaline solution without adding any toxic substances, and eventually grow into a rod by winding around a needle-shaped ZnO core, controlling even formation. Another goal was to identify the surface configuration that best impacts the antibacterial activity of ZnO nanomaterials against Gram-positive and Gram-negative bacteria. Eventually, we confirmed how antibacterial activity varies depending on the defects in the ZnO crystal structure, photoabsorption, light-induced charge separation, and photoluminescence characteristics. Additionally, ZnO nanomaterials were measured for the strength of their radicals (∙OH and ∙O_2_^−^) formed by oxidation and reduction reactions, in order to determine which reaction enhanced their antimicrobial properties. We intend to provide researchers seeking to synthesize metal oxide particles with excellent antibacterial properties in the future with the most optimal properties of ZnO surfaces and a rational manufacturing method for this study.

## 2. Materials and Methods 

### 2.1. Materials and Reagents

Zinc acetate dihydrate (Zn(CH_3_COO)_2_·2H_2_O, ACS reagent, ≥98%, Sigma Aldrich, St. Louis, MO, USA) and sodium hydroxide (NaOH, purity 97%, Junsei, Tokyo, Japan) as reagents used to synthesize ZnO in this study. Methyl blue ([[4-[bis [4-[(sulfophenyl)amino]phenyl]methylene]-2,5-cyclohexadien-1-ylidene]amino]-benzenesulfonic acid disodium salt, (C_37_H_27_N_3_Na_2_O_9_S_3_)) was purchased from Sigma Aldrich. All the chemical materials were of analytical grade and were used without further purification.

### 2.2. Synthesis of ZnO at Low Temperatures and Strong Alkaline Conditions

**Scheme 1:**
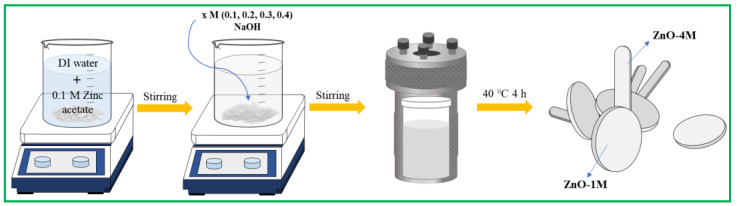
Synthesis process diagram of ZnO nanomaterials via the modified sol–gel method.

ZnO was synthesized using a modified sol–gel method, as shown in Scheme 1. Zinc acetate was dissolved in deionized water and stirred at room temperature for 1 h. NaOH (1.0, 2.0, 3.0, or 4.0 M) was added to this solution and continuously stirred for 1 h until homogenous. The solutions turned white in color and were moved to the autoclave to be thermally treated at 40 °C for 4 h. Finally, four types of ZnO powders were obtained: ZnO–1M, ZnO–2M, ZnO–3M, and ZnO–4M.

### 2.3. Characterizations

The crystal structures and morphologies of the synthesized materials were identified using X-ray diffraction (XRD, Miniflex 600, Rigaku, Cu Kα source, λ = 0.15418 nm, 40 kV, 15 mA). The presence of organic molecules in the samples was characterized by Fourier-transform infrared (FTIR) spectroscopy (Nicolet iS10 spectrometer, Thermo Fisher Scientific, Waltham, USA). The FTIR spectra were recorded as the average of 32 time scans in the range of 400–3600 cm^−1^. The diffuse reflectance spectroscopy-ultraviolet (DRS-UV)-visible spectra of the pelleted samples were confirmed using a Scinco spectrophotometer connected to a DR accessory in the range of 200–800 nm using BaSO_4_ as the background. Photoluminescence (PL) spectra were measured using a PerkinElmer fluorescence spectrophotometer with a He–Cd laser source with an excitation wavelength of 320 nm. Time-resolved photoluminescence (TRPL) measurements were performed using a confocal microscope (MicroTime 200; PicoQuant GmbH, Berlin, Germany) with an excitation wavelength of 360 nm. The decay curves were fitted using a bi-exponential decay function to deconvolute the instrument responses. The photocurrent density was measured using a Sun 2000 solar simulator (IviumStat, ABET Technologies, 620 nm LED, 298 K, 0.5 mW, five cycles). Mott–Schottky analysis was performed using a potentiostat/galvanostat equipped with a CompactStat (IVUIM Tech., Eindhoven, The Netherlands) at frequencies of 1000, 3000, and 5000 Hz within a DC potential range of 0–1 V.

### 2.4. Test Methods for Determining Photocatalytic Activity and Antibacterial Assays Conducted

**Scheme 2:**
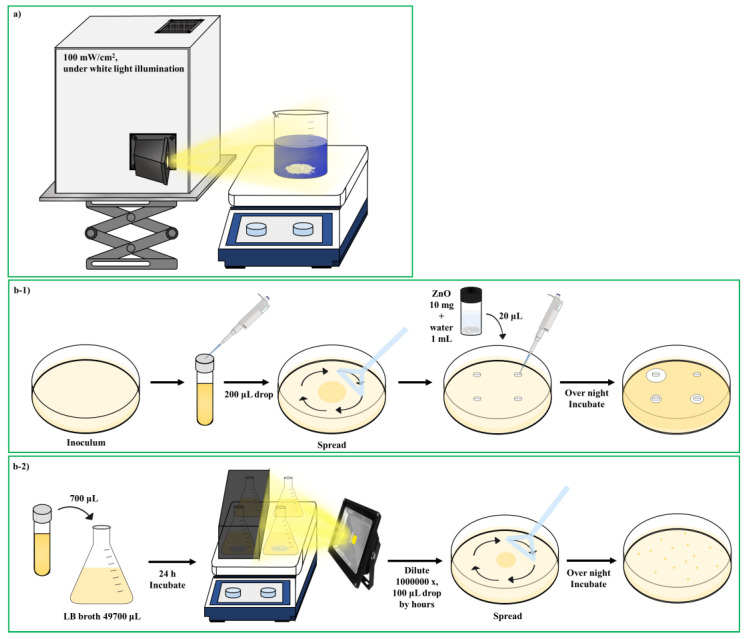
(**a**) Photolysis system for MB dye. (**b**) Schematic diagram of the antibacterial test process. (**b-1**) Disc diffusion method. (**b-2**) Antibacterial test in LB broth.

The photocatalytic activity of ZnO was measured in a photoreaction system using the degradation of methyl blue (MB) at room temperature under a 100 mW/cm^2^ xenon lamp with wavelength in the one sun region as shown in Scheme 2a. Next, 1 g/L of a photocatalyst is added to MB at a concentration of 100 mg/L. Thereafter, the solution was placed in the dark for 30 min to reach the adsorption–desorption equilibrium of the dye on the ZnO surface before irradiation. The suspension was then exposed to a xenon lamp, at a reactor-to-lamp distance of 5.0 cm, to degrade the MB dye. During the reaction, the solution was continuously stirred. Each sample was removed at every 1 h time interval and centrifuged at 5000 rpm for 20 min to remove the photocatalyst particles for analysis. For optical measurement of remaining MB dye, the dye was diluted (1/10 times) with deionized water. Finally, the absorbance of the MB dye in the supernatant was recorded using a DRS-UV spectrophotometer at the maximum absorption wavelength of the dye. Before the test, the concentration of MB was calculated based on calibration at a wavelength (λ_max_) of 650 nm. The dye removal rate (η) was calculated as follows [29]:

η = (C_0_ − C_t_)/C_0_ × 100%

where C_0_ and C_t_ are the MB dye concentrations after self-photodegradation and the different irradiation times, respectively.

Synthesized ZnO nanomaterials were evaluated for their antimicrobial activity against the respective representative Gram-positive bacteria *(Bacillus manliponensis*, *Micrococcus luteus*, *Staphylococcus aureus)* and Gram-negative bacteria *(Escherichia coli).* The antibacterial activities of the ZnO nanomaterials were first assessed using Bauer Kirby’s agar disk diffusion method against various bacterial species (*M. luteus*, *B. manliponensis*, *E. coli*, and *S. aureus*), as shown in Scheme 2b-1. Luria-Bertani (LB) agar was weighed and dissolved in a warm water bath. Afterward, it was sterilized for 15 min at 121 °C and 15 lbs pressure in an autoclave. Once the sterilized medium was cooled to 50 °C, 20 mL of the medium was poured into each sterilized Petri dish under aseptic conditions and then allowed to solidify under laminar airflow. LB broth was inoculated with pure *M. luteus* and *B. manliponensis* cultures and kept in a rotary shaker at 200 rpm for 24 h at 37 °C. The number of cells was adjusted to ~1 × 10^6^ CFU/mL and swabbed onto LB agar plates. Sterile paper disks containing the ZnO sample (10 mg) dissolved in water were placed on each plate and incubated again at 37 °C for 24 h. The antibacterial activity was assessed by measuring the zone of inhibition (ZOI). The experiments were done in triplicate and the ZOI diameter values were averaged.

Second, as shown in Scheme 2b-2, to quantitatively analyze the effect of ZnO on bacterial cell overnight culture, the test bacteria were used to inoculate LB media (~10^6^ CFU) with and without the addition of the synthesized nanomaterials. The culture flasks were placed in a shaker incubator (200 rpm) for 0, 1, 2, or 3 h at 37 °C. In addition, the same actions were performed while irradiating with LED (600 nm) light. After the specified time, samples were drawn and serially diluted. The dilutions were spotted onto Muller Hinton Agar plates, and after 24 h of incubation, colonies were counted in terms of CFU. An uninoculated medium containing nanomaterials was used as a negative control. The sterilization effect was calculated using the following formula [30].

C(%) = [(A − B)/A] × 100%

where C is the antibacterial and bactericidal effects, A is the average number of colonies formed in the control group (CFU/mL), and B is the average number of colonies formed in the experiment (CFU/mL).

### 2.5. ROS Generation Experiment

To further study the photocatalytic mechanism, the main reactive species (radicals and holes) were detected using radical-scavenging experiments. DMPO (5,5-dimethyl-1-pyrroline N-oxide) was used as a free-radical trapping agent to detect ∙OH and ∙O_2_^−^ radicals, ppm and spin-trapping ESR analysis was performed. The measurements were obtained by adding 10.0 mg of sample and 40 μL-DMPO to 1.0 mL-H_2_O_2_/9.0 mL-H_2_O and 10 mL-CH_3_OH.

## 3. Results and Discussion

### 3.1. Physicochemical Characterization

**Figure 1:**
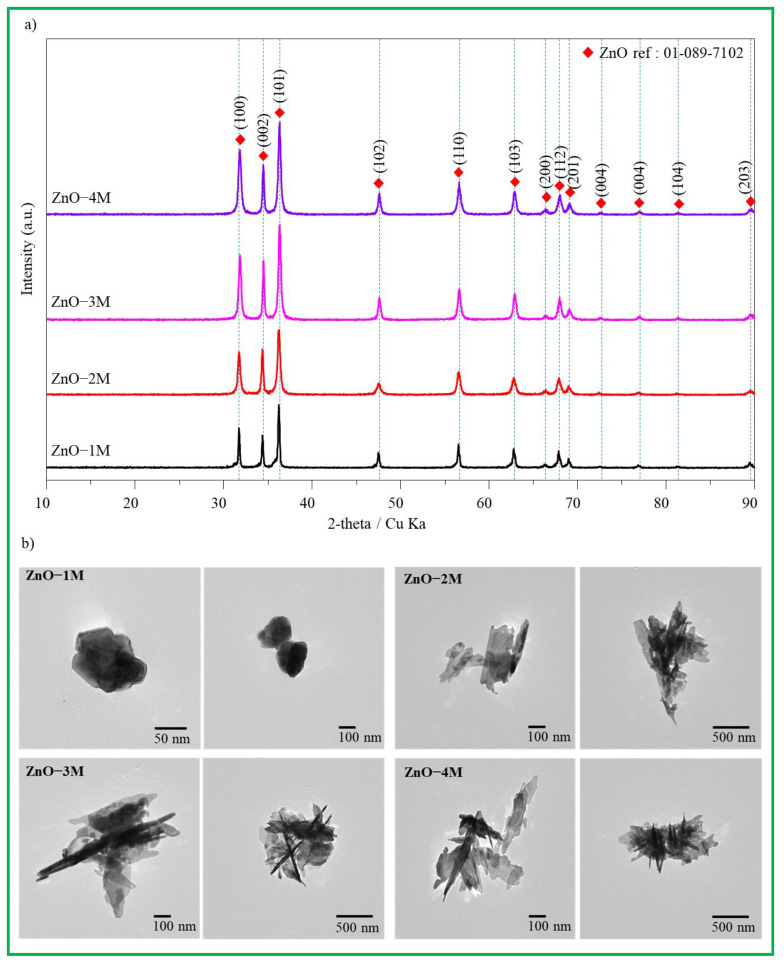
(**a**) XRD patterns of synthesized ZnO particles. (**b**) TEM images of synthesized ZnO particles (ZnO–1M, ZnO–2M, ZnO–3M and ZnO–4M) at two different magnifications.

Figure 1a illustrates the XRD patterns of ZnO prepared in NaOH solution at 40 °C using a modified sol–gel process. The crystallinity of the samples corresponded to hexagonal wurtzite-structured zinc oxide particles, and the diffraction data agreed well with the standard card JCPDS 01-089-7102 [31]. The characteristic crystalline reflections of ZnO at 31.81, 34.44, 36.31, 47.60, 56.62, 63.01, 66.48, 67.97, and 69.19° corresponded to the (100), (002), (101), (102), (110), (103), (200), (112), and (201) planes, respectively. No reflections of other phases were detected, indicating the high purity of the prepared samples. Additionally, as the concentration of NaOH increased, the intensities of the peaks increased, and the widths of the peaks became broad. The crystalline sizes of the samples were calculated using the Scherrer equation [32] based on the (101) plane; the crystal sizes of each sample were 22, 18, 19, and 19 nm for ZnO–1M, ZnO–2M, ZnO–3M, and ZnO–4M, respectively. The crystallite sizes were slightly small for the ZnO nanomaterials obtained in strongly alkaline solutions.

In the TEM image in Figure 1b, the particle shape of ZnO–1M synthesized in the presence of 1.0 M NaOH was nearly circular shape. This implies that the Na^+^ ions of NaOH did not act as a capping agent and hydrolysis occurred in all axial directions, similar to the x-, y-, and z-axes, and crystals were grown. However, it can be assumed that from the needle-shaped ZnO nanomaterials obtained in the 2.0 M NaOH solution, Na^+^ ions acted as a capping agent, bound with the x- or y-axis of Zn, and interfered with the hydrolysis process. Moreover, as the NaOH concentration increased further, the needle thickness also increased and the particle grew into a rod shape. In particular, sheet-type ZnO was found to coexist. This image shows that in the presence of a strong alkaline solution, ZnO nanomaterials partially dissolve in a strong base, spread similar to a sheet, and finally grow into rods while winding the needle shaped ZnO core [33].

**Figure 2:**
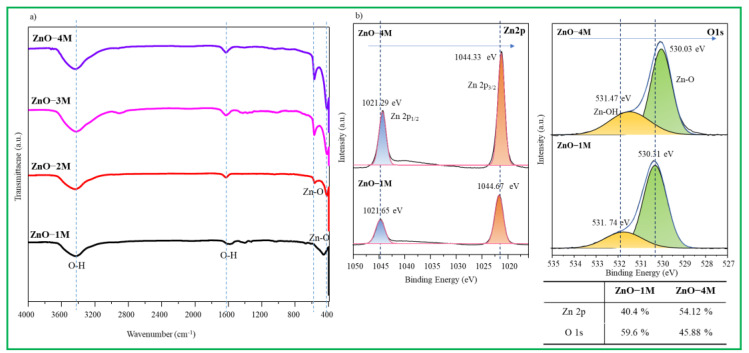
(**a**) FTIR spectra of synthesized ZnO particles. (**b**) XPS spectra of synthesized ZnO particles.

Figure 2a shows the FTIR spectra of the synthesized ZnO. In all samples, the band located between 421 and 559 cm^−1^ corresponds to the Zn-O stretching mode that can confirm the formation of ZnO [34], and the intensity of this peak increased in ZnO synthesized in a strong alkaline solution. In contrast, the presence of OH-bending and stretching vibration peaks were clearly shown at 1610 cm^−1^ and 3349 cm^−1^ [35], and these peaks were also stronger in ZnO synthesized in strong alkaline solution. This corresponds to Zn(OH)_2_ or Zn(OH)_4_^2−^, which is also related to the surface defects of ZnO and may affect the catalytic performance. Al-Gaashani et al. reported that Zn(OH)_4_^2−^ ions in strong bases react with OH groups on the surface of ZnO crystals and grow in the c-axis direction to synthesize a rod-like shaped ZnO particle [36].

In Figure 2b, high-resolution XPS spectra of Zn 2p and O 1s in the ZnO–1M and ZnO–4M particles are compared. The Zn 2p peak in the ZnO–1M nanomaterials was separated into two peaks located at approximately 1044.7 and 1021.7 eV attributable to Zn 2p_1/2_ and Zn 2p_3/2_, respectively, and adjusted with Gaussian fitting. The binding energy difference between Zn 2p_1/2_ and Zn 2p_3/2_ is 23 eV. Based on the results of other studies, it is considered to be a well-made ZnO [37]. The Zn 2p peak in the ZnO–4M particles shifted toward a slightly lower binding energy, and the ZnO surface was slightly reduced by the strong alkalinity. Meanwhile, the O 1s peaks, located at 530.3 and 531.7 eV, correspond to ∙O_2_^−^ in ZnO and Zn-OH, respectively [38]. In the ZnO–4M particle, the peaks shifted to somewhat lower energy, and in particular, the peak of Zn-OH significantly increased. This is related to the adsorbed OH^−^ groups on the surface of the ZnO nanomaterials or surface defects [39]. In contrast, the ratios of Zn:O in ZnO–1M and ZnO–4M particles were 40.4:59.6 and 54.12:45.88, respectively. The quantitative analysis of both samples revealed that approximately 10% of Zn defects occurred in ZnO–1M particles, while approximately 5% of O vacancies occurred in ZnO–4M particles.

**Figure 3:**
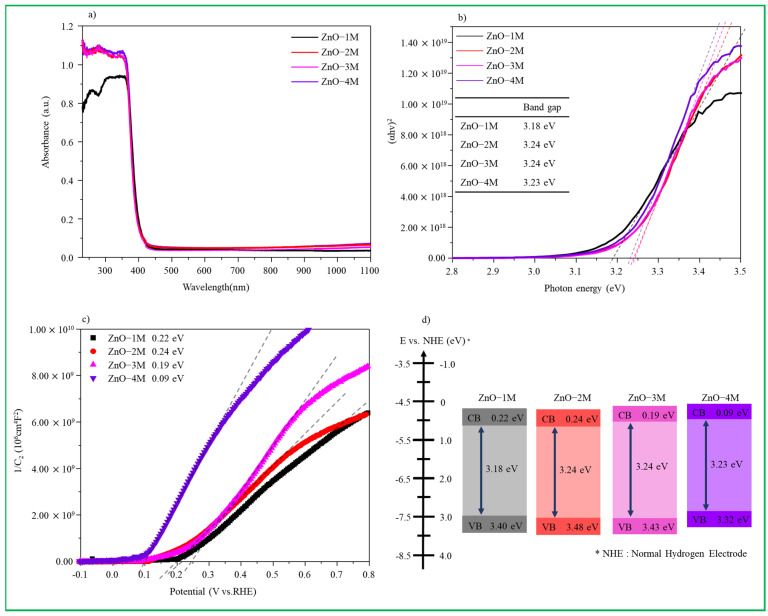
(**a**) DRS-UV-Visible absorption spectra, (**b**) Tauc’s plot curves, (**c**) Mott–Schottky’s plot curves, and (**d**) energy diagrams for the bandgap positions of the samples.

In Figure 3a, the DRS-UV-visible absorbance peaks in the ZnO nanomaterials are maximum at approximately 380 nm, and their optical bandgap energies (E_g_) were estimated using Tauc’s plot [40]. The plot of (αhv)^2^ versus the photon energy (h*v*) of the ZnO nanomaterials has a linear region and extrapolates a straight line to zero absorption. As shown in Figure 3b, for ZnO–1M, ZnO–2M, ZnO–3M, and ZnO–4M, they were calculated as 3.18, 3.24, 3.24, and 3.23 eV, respectively, and the difference in the band gap is related to the electron confinement phenomenon owing to the shape [41]. In contrast, Figure 3c shows the Mott–Schottky plot, where the flat band potential is obtained by extrapolating the curve to the x-axis, where the voltage of 1/C_2_ = 0. The flat band potentials obtained for ZnO–1M, ZnO–2M, ZnO–3M, and ZnO–4M were 0.22, 0.24, 0.19, and 0.09 eV, respectively, which were confirmed to be n-type semiconductors with positive slopes [42]. These values corresponded to the conduction band (CB) energy positions, and subtracting the bandgap from the CB energy position obtained from the Mott–Schottky plot yielded the valence band (VB) energy position. From this calculation, we could draw energy diagrams for the positions of all CBs and VBs in ZnO, as shown in Figure 3d. In the ZnO–4M particles synthesized in the strongest alkaline solution, the VB and CB energies increased. This may be related to the presence of oxygen vacancies.

### 3.2. Evaluation of Photoactivity and Antibacterial Property

The rapid development of industries leads to the discharge of industrial effluent into water bodies without taking appropriate precautions, endangering aquatic life and plants. Organic dyes are toxic, carcinogenic, and potentially mutagenic, yet their structures prevent their breakdown in the environment. MB dye exposure in humans may result in dizziness, vomiting, shock, paralysis, cyanosis, and neurological injuries. The wastewater is also discharged into water reservoirs, resulting in the contamination of water by pathogens like Staphylococcus aureus (*S. aureus*) and Escherichia coli (*E. coli*) in stagnant water storage facilities. It is therefore necessary for researchers to devise effective strategies to rid the environment of hazardous contaminants. There are many reports available where ZnO NPs are used only for single application with less activity or high activity only on low concentration of dye (1–30 ppm). In the present project, we have performed dual applications for antibacterial activity and photocatalytic degradation of high-concentration MB dye (100 ppm) for the first time.

**Figure 4:**
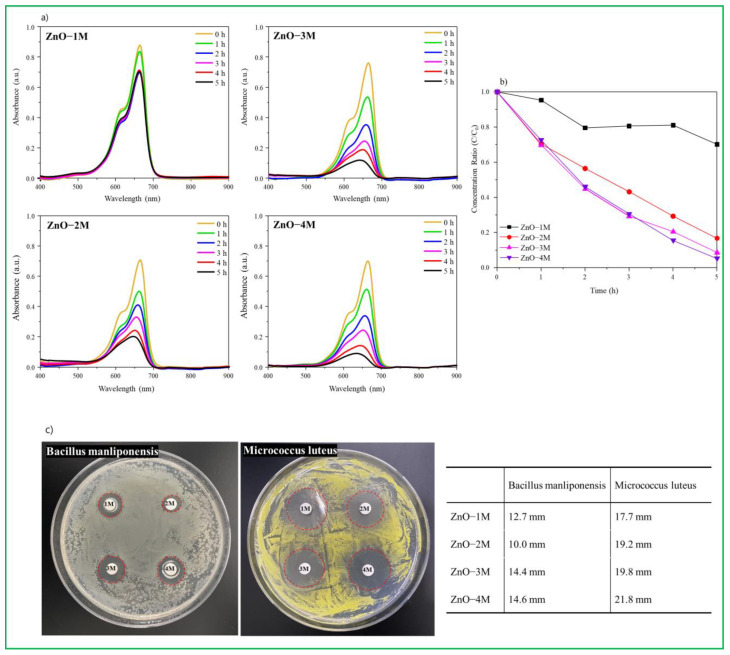
(**a**) UV-visible absorption intensity of MB decomposed with time. (**b**) Photodegradation performance of MB dye according to the catalyst; (**c**) antibacterial ability (zone of inhibition) against *B. manliponensis* and *M. luteus* bacteria on each catalyst. Inset table shows the antibacterial activity.

To determine the degradation activity of MB dyes under light condition the initial concentration of MB was fixed at 100 ppm, and MB decomposition experiments were performed per hour for each catalyst, as shown in Figure 4a. By observing the decrease in the peak at the absorption region over time, photocatalytic degradation of MB dye can be observed (600–700 nm). In the ZnO–1M particles, photocatalytic degradation of MB dye hardly proceeded after 2 h, and even after 5 h, the absorption peak of MB remained large. However, for the ZnO–2M particles, the absorption peak of MB was significantly reduced over time. In particular, in the ZnO–4M particles, photocatalytic degradation of MB dye took place more rapidly, and its color almost disappeared after 5 h. UV vis spectra shows the decrease in absorption of MB concentration with time (0–5 h) shown in Figure 4b, which indicates the complete degradation of MB dye. The MB dye decomposed more rapidly in the ZnO–4M particles made by the stronger alkaline condition. From the results, it can be noted that synthesized ZnO nanoparticles showed good photocatalytic activity against MB dye under the visible light and results are compared with earlier reports (Table 2). This is because MB, dye with the C_37_H_27_N_3_Na_2_O_9_S_3_ formula, is ionized into the 2Na^+^ cation and C_37_H_27_N_3_O_9_S_3_^2−^ anions in water, and 2Na^+^ is adsorbed as an electron scavenger on the basic ZnO surface, in particular, Zn(OH)_2_ or Zn(OH)_4_^2−^. Thus, more ∙OH radicals are activated on the holes of the VB, which can be used to decompose the C_37_H_27_N_3_O_9_S_3_^2−^ anion [43].

The antibacterial activity of ZnO nanomaterials was screened against the *B. manliponensis* and *M. luteus* (Gram-positive bacteria). The zone of inhibition (ZOI) is shown in Figure 4c and the antibacterial activity is summarized in inset table of Figure 4. The antimicrobial array disks clearly showed a region of inhibition around the four types of ZnO nanomaterials. The inhibitory diameters (ZOI) against *B. manliponensis*, were 12.7, 10.0, 14.4, and 14.6 mm for ZnO–1M, ZnO–2M, ZnO–3M, and ZnO–4M, respectively. The inhibitory diameters (ZOI) against *M. luteus*, were 17.7, 19.2, 19.8, and 21.8 mm for ZnO–1M, ZnO–2M, ZnO–3M, and ZnO–4M, respectively. The suppression diameter had an average deviation of ±2 mm. All ZnO nanomaterials showed higher antibacterial activity against *M. luteus* rather than *B. manliponensis*, and ZnO–4M synthesized in strong alkaline conditions exhibited a stronger antibacterial performance.

**Figure 5:**
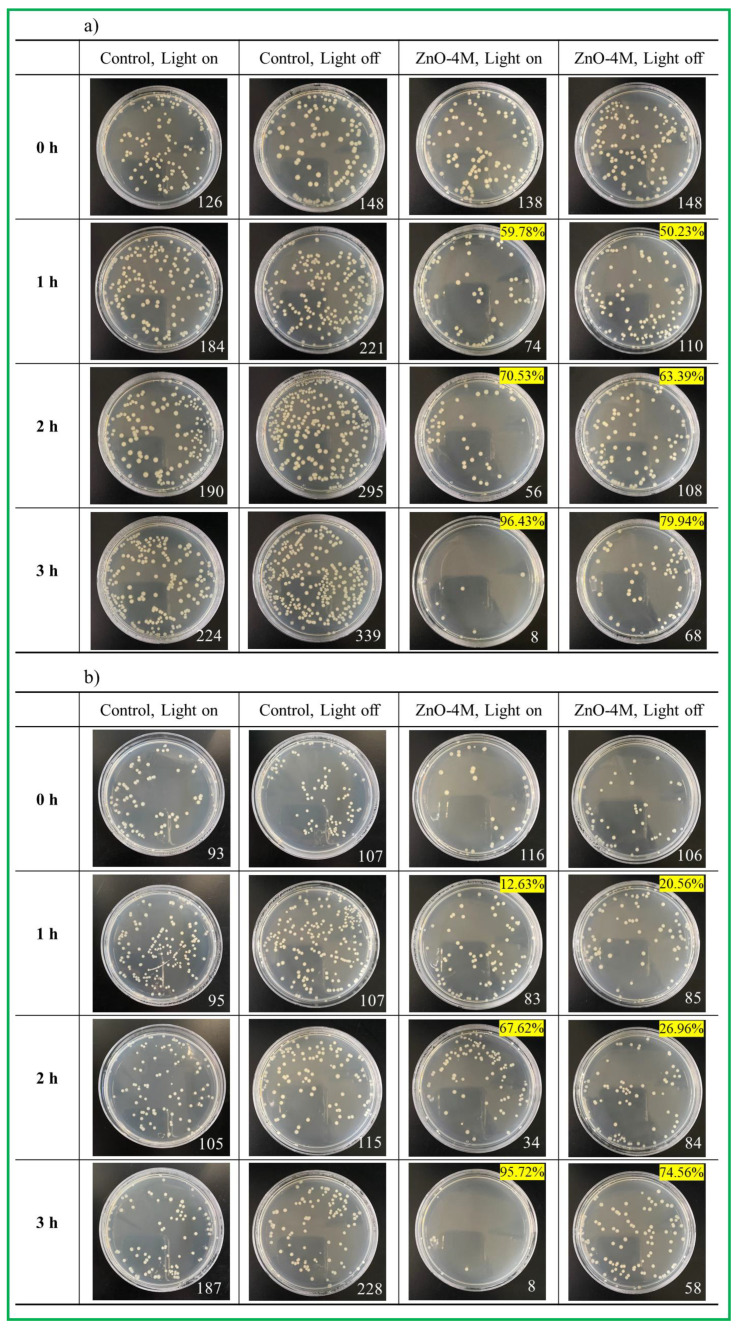
Sterilization capacity of ZnO–4M catalyst against (**a**) *S. aureus*; and (**b**) *E. coli*.

Assay for the ZnO–4M particles, which showed the best performance, is shown in Figure 5. First, with respect to the control group (where there was no catalyst), the bacteria grew stably when irradiated and non-irradiated with light. In particular, it was found that in the control group, the fertility of *S. aureus*, a Gram-positive bacterium, was stronger than that of *E. coli*, a Gram-negative bacterium. Meanwhile, we inferred that the bacteria were partially sterilized by light only as their growth rate was lower when irradiated compared to that when non-irradiated. In contrast, with respect to ZnO–4M particles, 79.94% sterilization performance was observed after 3 h when no light was irradiated, and 96.43% bactericidal activity was observed when irradiated with light for *S. aureus*. For *E. coli*, 74.56% sterilization performance was observed after 3 h when no light was irradiated, and 95.72% bactericidal activity was observed after 3 h of light irradiation. This result illustrated that Gram-positive bacteria (thin cell wall) were much easier to sterilize than Gram-negative bacteria (thick cell wall) [49], and the sterilization effectiveness increased significantly when irradiated with light compared to that seen with no light irradiation. Ultimately, these results show that ROS, such as ∙OH or ∙O_2_^−^ radicals induced on the ZnO surface by light, affect DNA destruction more effectively than ZnO nanomaterials. Antimicrobial mechanisms of ZnO have been reported. There are three antibacterial mechanisms of nanomaterials: there are three mechanisms for the antimicrobial activity of nanomaterials: release of antibacterial ions such as Zn^2+^, strong interaction between nanomaterials and microorganisms, and ROS formation by light irradiation [50]. Perhaps our results can be explained by focusing on the third mechanism. That is, the antibacterial mechanism in this study is suggested to be caused by ROS generation, and as the pH increases, the OH^−^ ions on the surface of ZnO increase and generate more ROS (∙OH or ∙O_2_^−^ radicals) [51], which in turn may lead to strong antibacterial properties.

The current results observed that in the presence of a strong alkaline solution, ZnO NPs partially dispersed into the strong base and eventually grew into a rod by winding a needle shaped ZnO core. The obtained synthetic ZnO NPs showed some spherical acicular ZnO core structures. These different peculiar morphologies showed distinct antibacterial effects against the target bacteria. It has been found to exhibit higher antibacterial activity against other bacteria under visible light or in the dark. It destroyed the bacterial membrane and released deadly active species. The ZnO NPs we synthesized exhibit excellent stability and with satisfactory feasibility for real wastewater, ZnO NPs can promote high-concentration wastewater treatment and disinfection. In fact, it was confirmed that we conducted anti-bacterial (*S. aureus* 96.43%, *E. coli* 95.72%) and high-concentration organic matter decomposition (MB dye 95%) tests with one single ZnO nanoparticle. This makes it possible for ZnO to be used for two applications simultaneously. A number of previous studies investigated the action of ZnO as an antibacterial agent or as a low-concentration organic matter decomposer. From the results, it can be noted that synthesized ZnO nanoparticles showed good photocatalytic activity against MB dye under the visible light and results are compared with earlier reports (Table 2). Apart from *E. coli* and *S. aureus*, we also tested ZnO NPs for their ability to inhibit *B. manliponensis* and *M. luteus*, which are bacteria isolated from tidal flat sediments. Therefore, it was confirmed that the ZnO nanoparticles synthesized at 4M exhibited excellent antibacterial activity against various bacteria as a whole and good photocatalytic activity against MB dye activity. In conclusion, it was confirmed that our ZnO nanoparticles can have higher activity for both applications than conventional ZnO NPs.

### 3.3. Optical Properties of ZnO Particles

**Figure 6:**
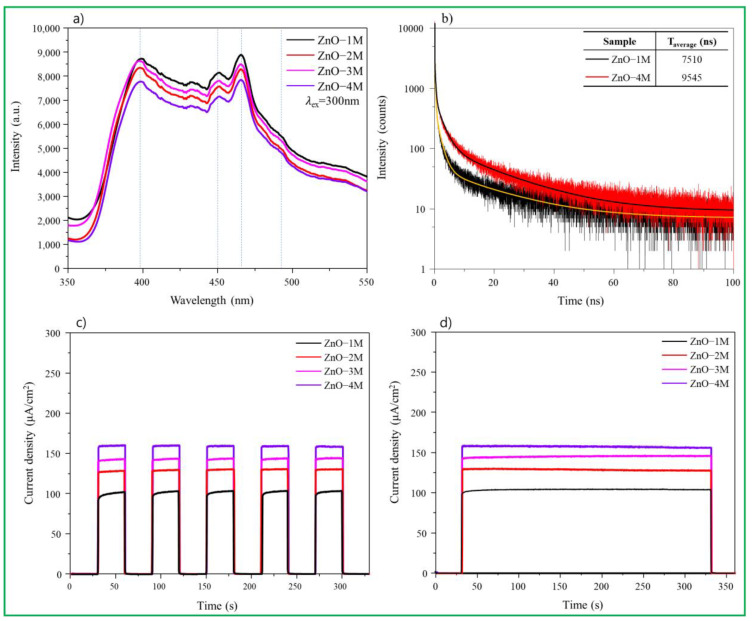
(**a**) PL curves. (**b**) Time-resolved PL curves for ZnO–1M and ZnO–4M particles. (**c**) Photocurrent density cycle curves. (**d**) Time-resolved photocurrent density curves of synthesized ZnO nanomaterials.

The PL spectrum is a powerful tool for investigating morphological features, defects, and chemical composition. As shown in Figure 6a, ZnO nanomaterials exhibit a large emission band in the UV region (near-band emission) at approximately 400 nm and a broad (deep) emission band in the visible region (440–500 nm) [52]. The strength, location, and proportion of PL depend on the structural characteristics of ZnO nanomaterials. There are major defects such as zinc vacancies, single- and double-ionized oxygen vacancies, neutral oxygen vacancies, and oxygen gaps [53]. Moreover, it was recently found that oxygen sites in ZnO particles cause changes in the antimicrobial properties of the nanomaterials [54]. The peaks observed at 450 and 470 nm are associated with oxygen vacancies and interstitial zinc ion defects, respectively, and the emission peak at 490 nm corresponds to the transition between photoexcited holes and single ionized oxygen vacancies. In the ZnO particles synthesized under strong alkaline conditions, the intensity of the peaks seemed to decrease; however, the peaks at 450, 470, and 490 nm were more clearly observed than the peak at 400 nm, suggesting that the number of oxygen vacancies was higher. In contrast, time-resolved photoluminescence in Figure 6b was performed to explain the exciton lifetime of ZnO. The ZnO–1M particles had a rapid decay constant, indicating that prompt recombination of photogenerated carriers from the CB to the VB occurred. Meanwhile, the slow decay in the ZnO–4M particles was due to the slow recombination of photogenerated carriers. This slow recombination may be due to oxygen defects acting as trapping centers, which can slow the relaxation of carriers before recombination. In Figure 6c, the intensity of the photocurrent density decreased in the order of the ZnO–4M, ZnO–3M, ZnO–2M, and ZnO–1M particles. When irradiated with visible light, ZnO–4M was the most stable and had the highest photocurrent density. This implies that the ZnO–4M particles show a better visible light response and more efficient charge separation than the other particles. Moreover, the time-dependent decay rate of the photocurrent density shown in Figure 6d remained stable for all particles up to 330 s and was highest for ZnO–4M particles.

**Figure 7:**
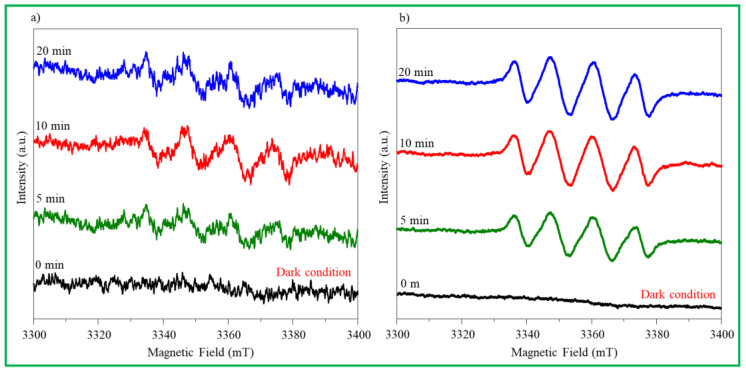
Spin-trapping ESR curves for ZnO–4M particle. (**a**) ∙OH radical, (**b**) ∙O_2_^−^ radical.

Spin-trapping ESR analysis was performed using DMPO as a free-radical trapping agent to estimate the charge transfer path in the ZnO nanomaterials. The ∙OH and ∙O_2_^−^ radicals were detected over time (0, 5, 10, and 20 min) and are shown in Figure 7a,b. First, after adding 40 mL of DMPO to an aqueous solution of 1.0 mL of H_2_O_2_ and 9.0 mL of H_2_O, 10.0 mg of a ZnO–4M sample was dispersed to measure ∙OH radical levels. As shown in Figure 7a, at first, the signal resembles background noise even under dark conditions; however, a minute signal for the DMPO-∙OH radical was detected. After 5 min of light irradiation, the signals appeared in a perfect peak, and the longer the light irradiation time, the stronger the signal intensity. This increase implies that when electrons present in the VB of ZnO–4M are excited to the CB in the presence of light, ∙OH radicals are generated in the VB holes, and the amount of ∙OH radicals generated increases over time. In contrast, in Figure 7b, the same amount of DMPO was added to 10 mL of CH_3_OH and 10.0 mg of a ZnO–4M sample was dispersed to measure the ∙O_2_^−^ radical. No signal was detected in the dark. However, the signal was detected when the light was irradiated. In particular, the DMPO-∙O_2_^−^ signal is observed more clearly than the DMPO-∙OH signal. Additionally, the signal intensity increased significantly with light irradiation time. This trend indicates that many electrons excited at the VB of the ZnO–4M particles were collected in the CB by light irradiation. The fact that the DMPO-∙O_2_^−^ signal is stronger than that of DMPO-∙OH after 20 min is evidence that, in the ZnO–4M particles, ∙O_2_^−^ radicals are more effectively generated than OH radicals. Partial defects such as Zn(OH)_2_ or Zn(OH)_4_^2−^ were present on the surface of ZnO–4M prepared in a strong alkaline solution, and the excitation acted as an H^+^ or Na^+^ trap and became a hole scavenger site. Moreover, oxygen defect sites in the lattice act as electron traps and attract electrons to promote charge separation. They are believed to act as a factor to increase the electron concentration in CB. In this study, oxygen defect sites and basic sites exist simultaneously on the surface of the ZnO nanomaterials synthesized in strong alkaline conditions, which simultaneously attract electrons and cations to facilitate charge separation and increase ∙O_2_^−^ radicals in the CB. Therefore, we believe that this largely improved the photoactivity and antibacterial performance. In contrast, as shown in Figure 5, even when no light was irradiated, the sterilization activity exceeded 75%, which was probably due to the ∙OH radicals generated in ZnO. As shown in Figure 7a, even under dark conditions, ∙OH radicals were generated in small amounts on the ZnO surface. It can be concluded that the antibacterial activity of ZnO is greatly affected by ROS generation, and it is more strongly affected by ∙OH radicals in the absence of light and by ∙O_2_^−^ radicals in the presence of light.

### 3.4. Mechanisms for Photocatalytic and Antibacterial Activity

**Scheme 3:**
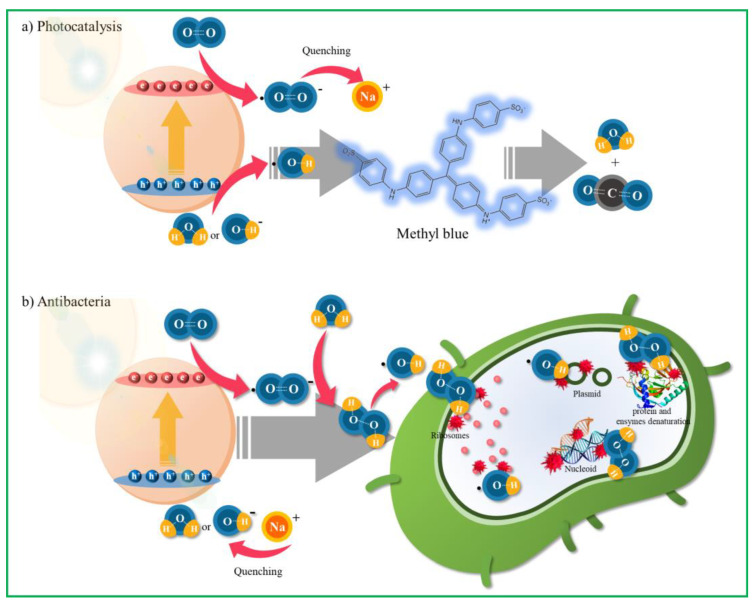
Photolysis and antibacterial mechanisms on the surface of ZnO–4M catalysts. (**a**) MB photolysis mechanism; (**b**) antibacterial mechanism.

Scheme 3 shows the mechanism of photocatalytic and antibacterial reactions in ZnO–4M. Photocatalytic reactions generally include photoexcitation, charge separation and transfer, and surface oxidation-reduction reactions [55]. To understand the mechanism of ZnO for MB dye degradation, it is necessary to determine the ROS that play an important role in the photocatalytic degradation process. Because we did not add a scavenger separately in this study, Na^+^ in MB dye likely acts as an ∙O_2_^−^ scavenger during the photolysis of MB dye to ZnO–4M. Therefore, in this study, the generated ∙O_2_^−^ radicals were inhibited by Na^+^ during the photocatalytic decomposition of MB dye to ensure that the ∙OH radicals become reactive species. Hence, in the photocatalytic decomposition mechanism of MB dye in ZnO photocatalysis under one-sun irradiation, electrons in the VB move to the CB under light irradiation, as shown in Scheme 3a. At this time, the VB holes of the ZnO–4M particles reacted with hydroxyl groups (OH^−^) or H_2_O to generate hydroxyl radicals (·OH). The photoelectrons transferred to the CB reduced the oxygen (O_2_) adsorbed on the photocatalyst surface to the ∙O_2_^−^ radical but were eventually combined with Na^+^ and quenched. Finally, the MB dye was decomposed by the ∙OH radicals generated in the VB. In contrast, a schematic diagram of the antimicrobial activity in ZnO–4M under one-sun light is presented in Scheme 3b. The excellent antibacterial performance of ZnO–4M is due to rapid charge separation by oxygen vacancies and the excessive generation of ROS by surface alkalinity. Generally, it has been reported that only H_2_O_2_ enters the cell surface due to the negatively charged nature of the bacterial cell surface [56]. However, the ZnO–4M particles prepared in this study exhibited excellent antibacterial properties against both Gram-positive and Gram-negative bacteria. Moreover, excessive ∙O_2_^−^ radical production was confirmed by the DMPO-ESR results. Therefore, on the surface of the ZnO–4M particles, the generation of ROS due to visible/ultraviolet radiation is considered to exhibit strong antibacterial performance. In particular, we concluded that the ∙O_2_^−^ radical generated on the surface of ZnO–4M is transformed into H_2_O_2_ via water adsorption and then converted to the ∙OH radical (or by itself) before attacking the DNA of bacteria.

## 4. Conclusions

This study aimed to investigate the defects in the ZnO crystal structure, light absorption capacity, light-induced charge separation, electron transfer rate, and photoluminescence characteristics and to investigate the correlation between them, antibacterial performance, and catalytic activity. Furthermore, the strength of ∙OH and ∙O_2_^−^ radicals generated on the surface of synthesized ZnO at various alkali concentrations were measured, and the oxidation and reduction reactions that would be more advantageous in enhancing antibacterial properties were determined. Although the shapes of the nanomaterials were different, they all exhibited lower-crystallinity blue emission spectra. In particular, in the ZnO–4M particles obtained from strong alkalis, oxygen depletion and surface OH groups increased simultaneously. Under light irradiation, ∙O_2_^−^ radical production increased more than that of the ∙OH radical, and this powerful radical effectively attacked both Gram-positive and Gram-negative bacteria. The ZnO–4M particles showed 96.43 and 95.72% sterilization effects against Gram-positive bacteria such as *S. aureus* and Gram-negative bacteria *E. coli* after 3 h, respectively.

## Figures and Tables

**Table 1:** Methods and conditions reported in the literature for the synthesis of ZnO.

Synthesis Method	Heat Treatment	Precursor	Ref.
Mechano- chemically	Calcination: 400–800 °C, 2 h	Zinc chloride anhydrous (ZnCl_2_·H_2_O), sodium carbonate anhydrous (Na_2_CO_3_·H_2_O), sodium chloride (NaCl)	[24]
Mechano- chemically	Calcination: 2 h, 600 °C	Zinc chloride anhydrous (ZnCl_2_·H_2_O), sodium carbonate anhydrous (Na_2_CO_3_·H_2_O), sodium chloride (NaCl)	[25]
Sol-gel	Reaction: 60 °C, Calcination: 500 °C	Zinc acetate dihydrate (Zn(CH_3_COO)_2_·2H_2_O), oxalic acid (H_2_C_2_O_4_), ethanol (C_2_H_6_O)	[26]
Sol-gel	Reaction: 50 °C, 1 h Calcination: 650 °C, 4 h	Zinc acetate dihydrate (Zn(CH_3_COO)_2_·2H_2_O), oxalic acid dihydrate (H_2_C_2_O_4_·2H_2_O), dichloromethane (CH_2_Cl_2_.), methanol (CH_3_OH), ethanol (C_2_H_6_O)	[27]
Solvo- thermal	Autoclave: 180 °C, 24–100 h	Trimethylamine N-oxide (C_3_H_9_NO), 4-picoline N-oxide (C_6_H_7_NO), Hydrochloric acid (HCl), toluene (C_7_H_8_), ethylenediamine (C_2_H_8_N_2_), N,N,N′,N′-tetramethylethylenediamine (C_6_H_16_N_2_)	[28]
Hydro- thermal	Autoclave: 40 °C, 4 h No calcination	Zinc acetate dihydrate (Zn(CH_3_COO)_2_·2H_2_O), sodium hydroxide (NaOH), DI water	This study

**Table 2:** Comparison of the photocatalytic degradation of MB under visible light using different catalysts.

Heat Treatment	Precursor	Degradation	Condition	Light Source	Ref.
Autoclave, 180 °C, 30 m	Zinc nitrate hexahydrate (Zn(NO_3_)_2_·6H_2_O), Sodium hydroxide (NaOH), DI water, Polyethylene glycol, Anhydrous ethanol	7 h MB 100%	MB (1.25 ppm), ZnO (2 g/L)	UV lamp (WFH-203, 220 V, 50 Hz),	[44]
Calcined, 500 °C, 3 h	Zinc nitrate hexahydrate (Zn(NO_3_)_2_·6H_2_O), Ammonia solution (NH_4_OH), silver nitrate (AgNO_3_), glacial acetic acid (CH_3_COOH), DI water	4 h MB 96%	MB (20 ppm), Ag-ZnO (1 g/L)	Xenon lamp of 100 W (one-sun region)	[45]
Calcined, 100 °C, 24 h	Zinc acetate dihydrate (Zn(CH_3_COO)_2_·2H_2_O), sodium hydroxide (NaOH), Ethanol (C_2_H_5_OH),	3 h MB 92%	MB (20 ppm), Fe-ZnO (1 g/L)	150 W mercury light (400–700 nm)	[46]
Calcined, 580 °C, 2 h	Zinc acetate dihydrate (Zn(CH_3_COO)_2_·2H_2_O), Cadmium acetate dihydrate (Cd(CH_3_COO)_2_·2H_2_O), Sodium lauryl sulfate (CH_3_(CH_2_)_11_OSO_3_Na), Sodium hydroxide (NaOH), DI water	2 h MB 95%	MB (6 ppm), Cd-ZnO (50 g/L)	Visible light irradiation	[47]
Autoclave, 150 °C, 24 h	Commercial nano zinc oxide (ZnO), Ammonia solution (NH_4_OH)	2 h MB 99%	MB (10 ppm) N-doped ZnO (2 g/L)	AM 1.5 simulated solar light	[48]
Autoclave, 40 °C, 4 h	Zinc acetate dihydrate (Zn(CH_3_COO)_2_·2H_2_O), Sodium hydroxide (NaOH), DI water	5 h MB 95%	MB (100 ppm), ZnO (1 g/L)	100 mW/cm^2^ xenon lamp (one-sun region)	This study
